# Comparison between Citral and Pompia Essential Oil Loaded in Phospholipid Vesicles for the Treatment of Skin and Mucosal Infections

**DOI:** 10.3390/nano10020286

**Published:** 2020-02-07

**Authors:** Iris Usach, Elisabetta Margarucci, Maria Letizia Manca, Carla Caddeo, Matteo Aroffu, Giacomo L. Petretto, Maria Manconi, José-Esteban Peris

**Affiliations:** 1 Department of Pharmacy, Pharmaceutical Technology and Parasitology, University of Valencia, Avda. V. Andrés Estellés, s/n Burjassot, Valencia 46100, Spain; iris.usach@uv.es (I.U.); elisabetta.margarucci@hotmail.it (E.M.); 2 Department of Scienze della Vita e dell’Ambiente, University of Cagliari, Via Ospedale 72, Cagliari 09124, Italy; mlmanca@unica.it (M.L.M.); caddeoc@unica.it (C.C.); matteo.aroffu@gmail.com (M.A.); manconi@unica.it (M.M.); 3 Department of Chemistry and Pharmacy, University of Sassari, Sassari 07100, Italy; gpetretto@uniss.it

**Keywords:** liposomes, pompia essential oil, citral, bacteria, yeast

## Abstract

*Citrus* species extracts are well known sources of bio-functional compounds with health-promoting effects. In particular, essential oils are known for their antibacterial activity due to the high content of terpenes. In this work, the steam-distilled essential oil from the leaves of *Citrus limon* var. *pompia* was loaded in phospholipid vesicles. The physico-chemical characteristics of the essential oil loaded vesicles were compared with those of vesicles that were loaded with citral, which is one of the most abundant terpenes of *Citrus* essential oils. The biocompatibility of the vesicles was assessed in vitro in human keratinocytes. Furthermore, the antimicrobial activity of the vesicles was tested while using different bacterial strains and a yeast: *Escherichia coli*, *Pseudomonas aeruginosa*, *Staphylococcus aureus*, and *Candida albicans*, respectively. The vesicles were small in size (~140 nm), slightly polydispersed (PI ~ 0.31), highly negatively charged (~ −73 mV), and able to incorporate high amounts of essential oil or citral (E% ~ 86%). Pompia essential oil and citral exhibited antimicrobial activity against all of the assayed microorganisms, with *P. aeruginosa* being the least sensitive. Citral was slightly more effective than pompia essential oil against *E. coli*, *S. aureus*, and *C. albicans*. The incorporation of citral in vesicles improved its antifungal activity against *C. albicans*.

## 1. Introduction

*Citrus limon* (L.) Osbeck var. *pompia* Camarda, which is also known as *Citrus* monstruosa, of the Rutaceae family [1], is an ancient endemic cultivar only grown in some warm areas of the north-eastern Sardinia (Italy) [2,3]. It is probably a hybrid species derived from citron and lemon that produces irregularly shaped, yellow fruits that are characterized by a low juice content and a reduced level of sugar [4]. An essential oil with antioxidant and antimicrobial activities can be obtained from pompia leaves. Limonene and citral (3,7-dimethyl-2,6-octadienal), which is a mixture of two isomeric acyclic monoterpene aldehydes, geranial (trans-citral or citral A) and neral (cis-citral or citral B), are the main components of pompia essential oil [3]. Citral, as well as essential oils containing citral and other terpenes, have been widely used in traditional medicine for the treatment of different infections, given their antimicrobial activity [5,6]. Recently, Zhang et al. (2020) [7] studied the antibacterial activity of seven essential oils that were obtained from Litsea cubeba, Rosemary, Citronella, Lavander, Spikenard, Bamboo leaf, and Vitex negundo. The most active oil against a food-derived strain (*Stenotrophomonas maltophilia*) was found to be Litsea cubeba oil. Such activity has been linked to the action of citral, which was the main component of the oil. The antibacterial activity of citral-containing essential oils is well documented in the literature, being in line with these findings [8,9].

The use of natural compounds is often limited because of their volatility and poor stability, despite the potential as therapeutic agents [10]. The incorporation in nanocarriers represents a suitable approach for overcoming these limitations. Liposomes are considered to be one of the most versatile nanocarrier systems capable of delivering both hydrophilic and lipophilic compounds. They offer several advantages as drug delivery systems, including high biocompatibility, self-assembly properties, the ability to carry large amounts of the payload, and a wide range of physico-chemical properties that can be modified in favour of their biological characteristics [11,12,13,14]. In previous studies, different essential oils were loaded in liposomes or modified liposomes to enhance their activities. For instance, cinnamon oil has been incorporated in soy lecithin, cholesterol liposomes to improve the antimicrobial activity [15]; *thymus* essential oil has been formulated in liposomes, glycerosomes, and Penetration Enhacer-containing Vesicles (PEVs) to enhance their antioxidant activity [16]; *Salvia tribola* and *Rosmarinus officinalis* essential oils have been loaded in liposomes, showing significant antioxidant, anti-inflammatory, and antibacterial activities, especially against *Klebsiella pneumoniae* [17].

In the present study, liposomes containing pompia essential oil or raw citral were prepared by direct sonication while using soy phosphatidylcholine. The physico-chemical properties of the obtained vesicles were evaluated, and their biocompatibility was assessed in vitro in human keratinocytes. The antibacterial and antifungal activities of the formulations were tested while using two gram-negative bacteria (*Escherichia coli* and *Pseudomonas aeruginosa*), one gram-positive bacterium (*Staphylococcus aureus*), and one yeast (*Candida albicans*).

## 2. Materials and Methods

### 2.1. Reagents

Lipoid S75 (S75), a mixture of soybean phospholipids (70% phosphatidylcholine, 9% phosphatidyletanolamine and 3% lysophosphatidylcholine), triglycerides and fatty acids, was purchased from Lipoid GmbH (Ludwigshafen, Germany). RPMI 1640 medium, DMEM medium, glucose, phosphate-buffered saline (PBS), tryptone water, methylene blue, dimethylsulfoxide (DMSO), and 3-(4,5-dimethylthiazol-2-yl)-2,5-diphenyltetrazolium bromide (MTT) were purchased from Sigma–Aldrich (Madrid, Spain). Mueller–Hinton agar and methanol were purchased from VWR Chemicals (Barcelona, Spain). Mueller–Hinton broth, 2-propanol, and hydrochloric acid 37% were supplied by Scharlau (Valencia, Spain). Sabouraud Dextrose Chloramphenicol Agar (SDCA) was obtained from Becton Dickinson (Madrid, Spain).

### 2.2. Antimicrobial Agents

Citral, gentamicin sulfate and clotrimazole were purchased from Sigma–Aldrich (Madrid, Spain). Pompia essential oil was extracted from pompia leaves that were collected in 2017 in Siniscola (Sardinia, Italy). 250 g of leaves were suspended in 700 mL of water and then subjected to steam distillation while using a Clevenger type apparatus for 2 h (European Pharmacopoeia 2002, Merk KGaA, Darmstadt, Germany). The yield of essential oil was 0.48% (v/w). The extraction was carried out in triplicate, and the obtained essential oils were collected separately, dried over anhydrous sodium sulphate (Na_2_SO_4_), and then stored under a nitrogen atmosphere at 4 °C in amber glass vials until their use and analysis.

### 2.3. Gas Chromatography-Mass Spectrometry (GC-MS) Analysis

The GC analysis of a solution of essential oil in n-hexane was carried out while using an Agilent 7890 GC (Palo Alto, CA, USA) that was equipped with a Gerstel MPS autosampler, coupled with an Agilent 7000C MSD detector as well as to a flame ionization detector (FID). Chromatographic separation was performed on a VF-Wax 60 m × 0.25 mm i.d., 0.5 µm film thickness column (Agilent). The following temperature program was used: 40 °C kept for 4 min., increased to 150 °C at a rate of 5.0 °C/min., kept for 3 min. then increased to 240 °C at a rate of 10 °C/min, and finally kept for 12 min. Helium was used as the carrier gas at a constant flow of 1.5 mL/min. The data was analyzed while using a MassHunter Workstation B.06.00 SP1, with identification of the individual components being performed by comparison with the co-injected pure compounds and by matching the MS fragmentation patterns and retention indices with the built-in libraries or literature data or commercial mass spectral libraries (NIST/EPA/NIH 2008; HP1607 purchased from Agilent Technologies (Palo Alto, CA, USA)). The relative percentages of the essential oil constituents were obtained by FID peak area normalization without the use of any correction factor.

### 2.4. Vesicle Preparation

Liposomes were prepared by hydrating S75 (60 mg/mL) overnight with water, in the presence of pompia essential oil or citral (20 mg/mL) at room temperature. After complete hydration, the samples were sonicated while using a high ultrasonic disintegrator (Soniprep 150, MSE Crowley, London, UK), three times for 15 cycles (5 s on and 3 s off; 13 microns of probe amplitude), to obtain small vesicles and homogeneous systems. The vesicle dispersions (2 mL) were purified from the non-incorporated active components by dialysis against water (4 L) at 25 °C for 3 h (with water refreshed every 30 min.) by using Spectra/Por^®^ membranes (12–14 kDa MW cut-off, 3 nm pore size; Spectrum Laboratories Inc., DG Breda, the Netherlands) [2,18]. The non-dialysed and dialysed vesicles were diluted with methanol and the amounts of citral or the main components of the pompia essential oil were quantified by gas chromatography, as reported in Section 2.3, to determine entrapment efficiency (EE), which was calculated as the percentage of the active components recovered in the dialysed samples versus the amounts that were found in non-dialysed samples.

### 2.5. Vesicle Characterisation

Transmission electron microscopy (TEM) confirmed vesicle formation and morphology. The samples were stained with 1% phosphotungstic acid aqueous solution and examined under a JEM-1010 (Jeol Europe, Paris, France) transmission electron microscope, at an accelerating voltage of 80 kV, equipped with a digital camera MegaView III and the “AnalySIS” software.

Photon Correlation Spectroscopy determined the average diameter and polydispersity index of the vesicles while using a Zetasizer nano (Malvern Instruments, Worcestershire, UK). The zeta potential of the vesicles was estimated while using the Zetasizer nano by means of the M3-PALS (Phase Analysis Light Scattering) technique. Prior to the analysis, samples (100 μL) were diluted with water (10 mL).

### 2.6. In Vitro Biocompatibility

The biocompatibility of the formulations was evaluated in vitro using immortalized human keratinocytes (HaCaT), which were grown as monolayers in 75 cm^2^ flasks that were incubated with 100% humidity and 5% CO_2_ at 37 °C. Dulbecco’s Modified Eagle Medium (DMEM) with high glucose, 10% foetal bovine serum, 1% penicillin/streptomycin, and 0.1% of fungizone, was used as culture medium. The cells were seeded into 96-well plates and then incubated for 24 h to reach subconfluence. Afterwards, the cells were treated for 48 h with citral or pompia essential oil in aqueous dispersion or incorporated in liposomes at different concentrations (20, 2, 0.2, and 0.02 μg/mL of pompia essential oil or citral). At the end of the experiment, the cells were washed with PBS, 100 µL of MTT [3(4,5-dimethylthiazolyl-2)-2,5-diphenyltetrazolium bromide] (0.5 mg/mL in PBS, final concentration) was added to each well to measure the percentage of live cells [19,20], and after 3 h, the formed formazan crystals were dissolved with DMSO, and the absorbance was spectrophotometrically read at 570 nm, which is the wavelength of maximum absorption of the formazan crystals, while using a microplate reader (Multiskan EX, Thermo Fisher Scientific, Inc., Walthan, MA, USA). The experiments were performed in triplicate and repeated at least three times. The results are shown as a percentage of cell viability in comparison to untreated cells (100% viability).

### 2.7. Microbial Strains

The antibacterial activity of pompia essential oil and citral, both in aqueous dispersion and loaded in liposomes, was tested against two gram-negative bacteria (*Escherichia coli* CECT 516 and *Pseudomonas aeruginosa* CECT 111), one gram-positive bacterium (*Staphylococcus aureus* CECT 239), and a yeast (*Candida albicans* CECT 1394). The cultures were kept at 36 °C ± 1 for 24 h. After 24 h of incubation, the bacterial and fungal suspensions were diluted with tryptone water to obtain an adequate density expressed as colony forming units per milliliter (CFU/mL).

### 2.8. Antimicrobial Tests

The antimicrobial activity of pompia essential oil and citral was evaluated while using both agar diffusion and microdilution tests, as described below.

#### 2.8.1. Agar Diffusion Method

Bacterial and fungal inocula were uniformly spread on the surface of a sterile Petri dish containing Mueller–Hinton agar while using sterile cotton swab. In the case of *C. albicans*, 2% of glucose and 0.5 μg/mL of methylene blue were added to the Mueller–Hinton agar. The addition of glucose provides better yeast growth, and methylene blue increases the definition of inhibition halos. Both of the supplements were incorporated during the preparation of the medium prior to sterilization. The density of the bacteria inoculum (*E. coli*, *P. aeruginosa* and *S. aureus*) was 1.4 × 10^8^ CFU/mL. In the case of *C. albicans*, the inoculum used was 6 × 10^6^ CFU/mL.

The paper discs were impregnated with 20 μL of each sample: pompia essential oil or citral solubilized in DMSO (300 mg/mL). The impregnated discs were placed onto the surface of the agar. The controls used were different as a function of the microorganism considered: gentamicin sulphate (1500 μg/mL in water) for bacterial strains and clotrimazole (250 μg/mL in methanol) for *C. albicans*. The plates were incubated at 36 °C ± 1 °C for 24 h, under aerobic conditions, and the diameter of the inhibition halo was measured in mm.

#### 2.8.2. Micro-Dilution Method

The broth micro-dilution method was applied while using 96-well microtitration plates and Eppendorf tubes. Microtitration plates and Eppendorf tubes were incubated at 35 °C for 24 h. Turbidity was measured in the case of microtitration plates, while MTT (3(4,5-dimethylthiazolyl-2)-2,5-diphenyltetrazolium bromide) was added to the Eppendorf tubes to colorimetrically determine the viable microorganisms.

A. Turbidimetric Assay

Microorganisms were seeded in microtitration plates and treated with pompia essential oil and citral at different concentrations (0.75, 1.5, 3, 6, and 12 mg/mL). Pompia essential oil and citral were first dissolved in DMSO (300 mg/mL) and subsequently diluted with Mueller–Hinton broth (for bacteria) or RPMI medium containing 2% glucose (for yeast) to obtain the desired concentrations.

In the case of the bacteria (*E. coli*, *P. aeruginosa* and *S. aureus*), the final inoculum concentration in the wells was approximately 5 × 10^5^ CFU/mL, while, in the case of *C. albicans*, the density of the inoculum was approximately 2 × 10^5^ CFU/mL. The 96-well plates were incubated at 35 °C for 24 h, and the turbidity in each well was measured with a spectrophotometer (λ = 530 nm) and compared with that obtained in the positive growth control wells (with inoculum and without the assayed substances) to determine the minimum concentrations of pompia essential oil and citral that are required to inhibit the growth of microorganisms by 50% (MIC_50_).

The minimum bactericidal concentration (MBC) or the minimum fungicidal concentration (MFC) were evaluated by spreading an aliquot (approx. 20 μL) of the content of each well on the surface of a Petri dish containing Mueller–Hinton agar in the case of bacteria (*E. coli*, *P. aeruginosa* and *S. aureus*) and SDCA for *C. albicans*, and measuring the lowest concentration of the samples with no growth of the tested microorganism after 24 h (bacteria) or 48 h (*C. albicans*) of incubation at 35 °C.

B. Colorimetric Assay

The MIC_50_ values for pompia essential oil and citral loaded liposomes were determined by means of a colorimetric method, as the standard turbidity test could not be used due to the self-turbidity of the liposomes. Briefly, liposomes that were diluted with Mueller–Hinton broth (for bacteria) or RPMI medium with 2% glucose (for yeast) were inoculated with the corresponding microorganism, while using the same volumes and inoculum concentrations that were described for the turbidimetric assay. Pompia essential oil and citral loaded liposomes were used at different concentrations: 0.15, 0.30, 0.60, 1.20, 2.50, 5, and 10 mg/mL. The Eppendorf tubes were incubated at 35 °C for 24 h, and at the end of the experiment 10 μL of MTT (5 mg/mL in H_2_O) were added to each tube. The tubes were incubated for an additional time period of 45 min. (bacteria) or 4 h (yeast). 1 mL of acidic isopropanol solution (0.4% (v/v) hydrochloric acid in isopropanol) was added to each tube to stop the MTT reduction by the microorganisms. The dispersions were centrifuged (8000× *g*, 3 min.) and the absorbance of the supernatant was measured at 570 nm, which is the wavelength of maximum absorption of the formazan crystals, while using a spectrophotometer (Multiskan EX, Thermo Fisher Scientific, Inc., Waltham, MA, USA) and compared with that obtained for positive growth control tubes (with inoculum and without liposomes) to determine the percentage of growth inhibition (MIC_50_).

### 2.9. Statistical Data Analysis

The results are expressed as means ± standard deviations. Multiple comparisons of means (ANOVA) were used to substantiate statistical differences between groups, while the Student’s t-test was used to compare two samples. Significance was tested at the 0.05 level of probability (*p* < 0.05). Data analysis was carried out with the XLStatistics software package for Excel [21].

## 3. Results

### 3.1. Essential Oil Characterisation

The phytochemical characterisation of pompia essential oil was carried out by liquid injection in a gas chromatographic device. Table 1 reports the main components of the essential oil and their relative percentages.

Limonene, lilalyl acetate, geranial, (E)-β-ocimene, linalool, and citral were the main components of the essential oil. Citral (the sum of geranial and neral) represents 17.9% of the essential oil and it is considered to be mainly responsible for the antimicrobial properties of the pompia essential oil [3].

### 3.2. Vesicle Preparation and Characterisation

**Figure 1:**
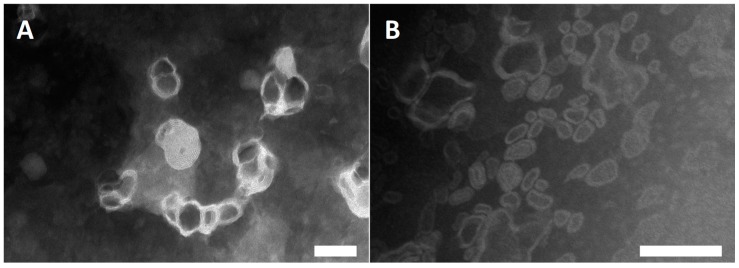
Transmission electron microscopy (TEM) images of pompia essential oil liposomes (**A**) and citral liposomes (**B**). Bars correspond to 100 nm.

The essential oil extracted from pompia leaves was loaded in liposomes, given that essential oils loaded liposomes are promising agents that can be used to increase the antimicrobial activity of essential oils [22]. The formulations were observed under TEM to confirm the formation of the vesicles and evaluate their structure and size (Figure 1). This technique was chosen, because the structure of soft matters is well preserved upon staining, avoiding artefacts and showing the real shape of the system.

The vesicles were small in size, irregularly shaped, and slightly aggregated.

Citral loaded liposomes were prepared as a reference. The empty liposomes were also prepared, in order to evaluate the effect of pompia essential oil and citral on the vesicle assembly. The empty vesicles were smaller (~75 nm) than vesicles loading pompia essential oil or citral (~152 and 129 nm, respectively), as reported in Table 2.

The incorporation of the essential oil or citral in liposomes led to a slight decrease in the homogeneity of the systems, as the polydispesity index increased from 0.28 to 0.31 (for pompia e.o. loaded liposomes) or 0.32 (for citral loaded liposomes). The formulations were reproducible and repeatable, despite the increase in size [23]. All of the vesicles were highly negatively charged, which is predictive of good stability on storage. Additionally, the incorporation of the payloads did not modify the surface charge of liposomes as compared to empty liposomes, thus confirming the localisation of the active compounds within the bilayer, as previously reported [24]. The amount of pompia essential oil and citral incorporated in the vesicles was very high (entrapment efficiency was around 86%), thus confirming the results of other authors [24].

### 3.3. In Vitro Biocompatibility Studies in Keratinocytes

**Figure 2:**
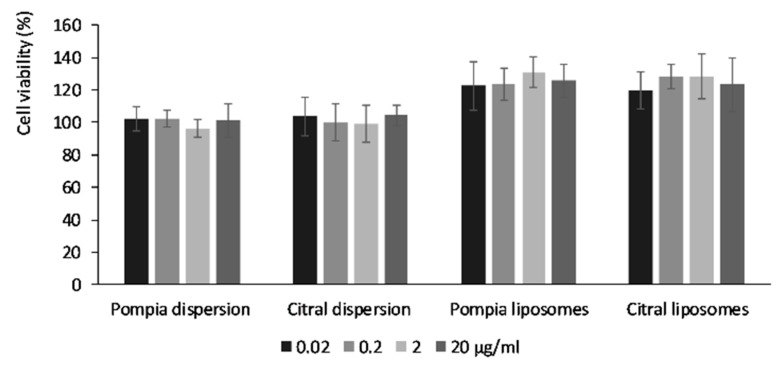
Cell viability of keratinocytes incubated for 48 h with pompia essential oil or citral loaded liposomes or the aqueous dispersions. Data are reported as mean values ± standard deviations of cell viability expressed as the percentage of control (100% of viability).

The biocompatibility of the formulations was evaluated by incubating keratinocytes with the prepared samples for 48 h. The dispersions of pompia essential oil or citral in water were used as references (Figure 2).

All of the formulations were highly biocompatible, as the cell viability was always above 100%. In addition, the incorporation of the essential oil or citral in the vesicles induced cell proliferation, with an increase in viability of ~20–30% in comparison with the untreated control cells (100% viability).

### 3.4. Agar Diffusion Method

The first screening was carried out by evaluating the efficacy of raw essential oil and citral against *E. coli*, *P. aeruginosa*, *S. aureus*, and *C. albicans*. The activity was compared with that of gentamicin and clotrimazole. The growth of *E. coli*, *S. aureus*, and *C. albicans* was inhibited by pompia essential oil and citral solutions (300 mg/mL in DMSO), confirming their ability to inhibit bacteria proliferation, as previously reported for pompia essential oil [3] and citral [5,25]. Very low inhibition halos were observed in the plates containing *P. aeruginosa* (Table 3).

Citral was more effective against *E. coli*, *S. aureus*, and *C. albicans*, and gave larger inhibition halos in comparison with that provided by pompia essential oil (Table 3). However, a dose of essential oil or citral 20 times higher had to be used (300 mg/mL) to reach an inhibition halo similar to that of gentamicin or clotrimazole.

### 3.5. Micro-Dilution Method

The MIC_50_ or MFC/MBC of pompia essential oil and citral solutions were measured while using the micro-dilution method (Table 4) [26].

The essential oil and citral showed comparable antimicrobial activities, although citral was slightly more effective (lower MIC_50_ and MFC/MBC) against *E. coli*, *S. aureus* and *C. albicans*. The antimicrobial results that were obtained while using liposomes were also similar, with citral loaded liposomes being more active than pompia liposomes against *E. coli*, *S. aureus* and *C. albicans*.

Unfortunately, it was not possible to measure the MIC_50_ and the MFC or MBC of pompia essential oil and citral when they were loaded in liposomes due the turbidity that was caused by liposome dispersions that altered the ABS values. For this reason, the ABS values were measured by means of a colorimetric assay [26].

The MIC_50_ and MBC against *P. aeruginosa* obtained while using pompia essential oil and citral in solution or loaded in liposomes were the same. Against *E. coli* and *S. aureus*, the pompia essential oil and citral solutions seemed to be active at concentrations lower than those that were found for the liposomes. The MIC_50_ against *C. albicans* obtained while using the essential oil in solution or loaded in liposomes were similar, while, in the case of citral loaded liposomes, the values were lower than those found for the citral solution, which points to the ability of liposomes to promote the delivery of citral. However, the MFC values were similar for both samples. These results suggest that the incorporation of citral in liposomes promotes the antifungal activity against *C. albicans*.

## 4. Discussion

The use of natural compounds as a safe and effective alternative to synthetic antimicrobials is mainly related to the large number of resistant strains to a wide variety of chemicals [27]. In particular, whole extracts from plants often provide greater in vitro and/or in vivo antimicrobial activity than the isolated constituents at an equivalent dose [28]. The antimicrobial activity of phytocomplexes can be attributed to a combination of complementary activities of different components [29]. Essential oils are complex mixtures of lipophilic, liquid, volatile, and often terpenoid compounds that are present in higher plants. Monoterpenes (hydrocarbon and oxygenated monoterpens), sesquiterpenes (hydrocarbon and oxygenated sesquiterpens), and, furthermore, phenolic compounds are mainly present [30]. Thanks to their chemical composition, essential oils possess antioxidant, anti-inflammatory, and antimicrobial activities [31]. Their antimicrobial activity is due to their ability to penetrate and interfere with the pathogen membrane, altering its structure and functionality, or interfering with the functionality of wall proteins that are involved in the processes of transport of compounds. Additionally, terpenoids have been found to interfere with the enzymatic reactions of energy metabolism [32,33].

Despite their high lipophilicity, the volatile components of essential oils are easily subjected to degradation, which is responsible for their reduced therapeutic effect. Additionally, essential oils display low in vivo bioavailability, due to their lipophilic nature [34,35].

Pompia essential oil was incorporated in liposomes in this study to prevent degradation and improve bioavailability. Liposomes that were loaded with citral, one of the main components of *Citrus* essential oils, were also prepared. Liposomes were prepared by direct sonication, avoiding the use of organic solvents and dissipative steps [36], according to the principles of Green Chemistry and ensuring the sustainability of the formulations [37]. The prepared vesicles were fairly spherical in shape, small in size, and able to incorporate high amounts of essential oil and citral. In addition, liposomes were highly biocompatible, as demonstrated by the in vitro studies that were carried out while using human keratinocytes. The ability of liposomes to improve the delivery of small and large molecules to the skin is well known [38,39]. Recently, they have been successfully proposed for the skin delivery of phytochemicals due to their high loading and carrier capabilities, as well as affinity with the skin [40]. The ability to potentiate the efficacy of essential oils was also demonstrated [41]. In previous studies, the optimal skin delivery performance of the phospholipid vesicles in the delivery of *Santolina insularis* essential oil were demonstrated [23]. Additionally, new biocarriers, called santosomes, were formulated by combining *Santolina insularis* essential oil and hydrogenated phosphatidylcholine. These vesicles were able to promote the skin delivery of phycocyanin, a cyanobacteria protein, thanks to the synergistic activity of the terpenes that are present in the essential oil and phospholipid [42].

Taking the recognized capabilities of liposomes into account to improve the dermal delivery of essential oils, in this study we focused on the evaluation of the antibacterial activity of liposomes that were loaded with pompia essential oil. The agar diffusion assay confirmed the antimicrobial activity of the oil against *E. coli*, *S. aureus*, *C. albicans*, and *P. aeruginosa* (Table 3); this being the first screening of antibacterial activity against different pathogens [43]. However, since microdilution and colorimetric assays are more accurate methods for the evaluation of the antibacterial activity of chemicals, they were also performed. These tests showed that pompia essential oil and citral were both active against all of the assayed pathogens and the incorporation in liposomes affected their inhibitory power differently (Table 4). The liposome formulations provided an increase of MIC_50_ and MBC against *E. coli* and *S. aureus*, but they did not modify the efficacy against *P. aeruginosa* and promoted the antifungal efficacy of citral against *C. albicans.* The obtained results confirm that citral is one of the most potent antibacterial components of pompia essential oil, and its incorporation in liposomes improves its antifungal activity.

## 5. Conclusions

The overall results suggest that pompia essential oil and citral can be suitably loaded in liposomes, thus facilitating their dermal delivery and interaction with epidermal cells. Subsequently, liposomes are able to increase the concentration of bioactive molecules in the infected target tissue and promote their efficacy. Citral loaded liposomes are more effective than pompia essential oil liposomes in counteracting the growth of bacteria (*E. coli* and *S. aureus*) and fungi (*C. albicans*).

## Figures and Tables

**Table 1:** Main compounds detected in the essential oil from pompia leaves.

Compound	RT	A%	RI
α-Pinene	13.37	0.4	1033
Camphene	14.85	tr	1081
β-Pinene	16.26	1.1	1126
Sabinene	16.67	0.4	1139
3-carene	17.59	0.9	1166
β-Myrcene	17.87	0.8	1175
2,3-Dehydro-1,8-cineole	18.89	tr	1206
limonene	19.24	29.7	1217
β-Z-Ocimene	20.26	0.5	1249
β-E-Ocimene	20.83	4.4	1267
p-Cymene	21.55	0.7	1290
Terpinolene	21.99	tr	1304
5-Hepten-2-one, 6-methyl-	23.43	0.9	1352
cis-Linalool oxide	26.27	0.1	1448
trans-Linalool oxide (furanoid)	27.05	0.0	1475
Citronellal	27.32	0.2	1484
Linalool	28.87	11.0	1535
Linalyl acetate	29.46	20.9	1555
3-Methoxy-p-cymene	30.73	0.2	1596
Terpinen-4-ol	31.07	0.3	1609
Megastigma-triene (not identified isomer)	31.40	0.7	1622
Neral	33.07	6.8	1689
α-Terpineol	33.17	2.2	1693
Neryl acetate	33.72	1.1	1718
Geranial	34.06	11.1	1735
Nerol	34.29	2.9	1746
cis-geraniol	35.00	0.5	1780
Geraniol	35.74	1.2	1819
Unknown	38.48	0.3	1985
Unknown	38.59	0.2	1992
Unknown	40.12	0.2	2090
Menthadien-1,2-diol	41.87	0.1	2216
Neric acid	42.90	0.2	2286

RT: retention time. RI: experimental retention indices calculated on a VF-Wax 60 m column; tr: trace. The results are expressed as relative percent area obtained by internal normalisation of FID chromatograms.

**Table 2:** Mean diameter (MD), polydispersity index (PI), zeta potential (ZP), and entrapment efficiency (EE) of empty, pompia essential oil-, and citral loaded liposomes. Mean values ± standard deviations were obtained from at least six replicates.

	MD (nm)	PI	ZP (mV)	EE (%)
Empty liposomes	75 ± 4	0.28 ± 0.02	−70 ± 2	-
Pompia e.o. loaded liposomes	152 ± 18	0.31 ± 0.05	−74 ± 5	85 ± 15
Citral loaded liposomes	129 ± 16	0.32 ± 0.05	−72 ± 4	88 ± 13

**Table 3:** Inhibition halo (IH) provided by pompia essential oil, citral, gentamicin and clotrimazole against *E. coli*, *P. aeruginosa*, *S. aureus*, and *C. albicans*. Mean values ± standard deviations (n = 4) are reported.

Compound (concentration)	*E. coli* IH (mm)	*P. aeruginosa* IH (mm)	*S. aureus* IH (mm)	*C. albicans* IH (mm)
Pompia e.o. (300 mg/mL)	13 ± 2	<5	25 ± 6	16 ± 3
Citral (300 mg/mL)	20 ± 4	<5	37 ± 1	25 ± 5
Gentamicin (1.5 mg/mL)	22 ± 4	24 ± 2	27 ± 2	-
Clotrimazole (250 µg/mL)	-	-	-	25 ± 1

**Table 4:** Values of MIC_50_ (minimum inhibitory concentration 50%) and MFC (minimum fungicidal concentration) or MBC (minimum bactericidal concentration) of pompia essential oil and citral in solution or loaded in liposomes. Results are expressed in mg of active ingredient per mL of final volume.

	MIC_50_ (mg/mL)	MFC or MBC
***E. coli***		
Pompia e.o.	3	12
Pompia e.o. loaded liposomes	10	>10
Citral	1.5	6
Citral loaded liposomes	5	10
***P. aeruginosa***		
Pompia e.o.	6	12
Pompia e.o. loaded liposomes	5	10
Citral	6	>12
Citral loaded liposomes	5	10
***S. aureus***		
Pompia e.o.	3	6
Pompia e.o. loaded liposomes	10	>10
Citral	1.5	3
Citral loaded liposomes	5	5
***C. albicans***		
Pompia e.o.	3	6
Pompia e.o. loaded liposomes	2.5	10
Citral	1.5	3
Citral loaded liposomes	0.6	2.5
